# Supplementary material for: Reduced Virus Load in Lungs of Pigs Challenged with Porcine Reproductive and Respiratory Syndrome Virus after Vaccination with Virus Replicon Particles Encoding Conserved PRRSV Cytotoxic T-Cell Epitopes
Source: Vaccines (Basel). 2021 Mar 2;9(3):208. doi: 10.3390/vaccines9030208 (PMC8000205; doi:10.3390/vaccines9030208)
Supplement: Supplementary file 1 [file vaccines-09-00208-s001.zip › SM files/Supplementary Data 1 ¿C Nucleotide and amino acid sequences of the VRPs.docx]

Supplementary data 1 – Nucleotide and amino acid sequences of the VRPs

Color codes:

restriction sites (ClaI: cATCGATg, KasI: GGCGCC, MluI: ggACGCGTg, NotI: GCGGCCGC)

C-terminal part of CSFV N^pro^

spacer

porcine UbV_76_

HA tag

Flag tag

Stop codon

(Poly)epitope

mutations

Backbone cassette (VRP 0)

Aa sequence (*NproC138A-UbV-HA-SIINFEKL-Flag):

GAILLKLAKRGEPRTLKWIRNFTDCPLWVTSCSGMQIFVKTLTGKTITLEVEPSDTIENVKAKIQDKEGIPPDQQRLIFAGKQLEDGRTLSDYNIQKESTLHLVLRLRGVYPYDVPDYAGASIINFEKLGRVSGDYKDDDDK*

Reverse translated and modified with flanking restriction sites:

CATCGATGGTGCCATACTGCTGAAGCTAGCCAAGAGGGGCGAGCCAAGAACCCTGAAGTGGATTAGAAATTTCACCGACTGTCCATTGTGGGTTACCAGTTGCAGCGGAATGCAGATTTTTGTCAAGACCCTCACCGGGAAGACCATTACACTGGAAGTGGAGCCAAGCGATACCATCGAGAATGTCAAGGCCAAGATCCAGGACAAGGAGGGCATCCCCCCAGACCAGCAGAGGCTGATCTTCGCTGGCAAGCAGCTGGAGGACGGACGCACCCTGTCCGACTACAACATCCAGAAGGAGAGCACCCTGCACCTGGTGCTGAGGCTGAGGGGCGTGTACCCATACGACGTGCCAGACTACGCTGGCGCCTCCATCATTAACTTTGAGAAACTCGGACGCGTGTCAGGAGACTACAAGGACGACGACGATAAATGAAGCGGCCGCT

VRP 1

Aa sequence of designed polyepitope (epitopes are underlined):

LSDSGRISYGDKKDPNISAVFQTYYNEQDQPTTMPSGFELYEDDQKDASDWFAPRYGDNPRTAPNEIAFGDKKDPRTAIGTPVYEEEDDQVYERGCRWYDQEEDGKIFRFGSHKWNDENPKVAHNLGFYF

Reverse translated and modified with flanking restrictions sites:

GCAGGCGCCCTCAGCGACTCTGGCAGGATTTCTTATGGGGACAAAAAGGACCCTAATATCTCCGCCGTGTTTCAGACCTACTACAACGAGCAGGACCAGCCCACCACCATGCCATCCGGCTTCGAGCTGTACGAGGACGACCAGAAGGACGCCAGCGACTGGTTCGCTCCAAGATACGGCGACAACCCCAGAACCGCCCCAAACGAGATCGCCTTCGGCGACAAGAAGGACCCAAGGACCGCTATCGGAACCCCAGTGTACGAGGAGGAGGACGACCAGGTGTACGAGAGGGGATGCAGGTGGTACGACCAGGAGGAGGACGGCAAGATCTTCAGGTTCGGCTCCCACAAATGGAACGATGAAAACCCCAAGGTCGCCCACAATCTCGGATTCTACTTCGGACGCGTGA

VRP 2

Aa sequence of designed polyepitope (epitopes are underlined):

NISAVFQTYYEQGEDGQVYERGCRWYDKPKKKKIFRFGSHKWGNGDGPKVAHNLGFYFNDDDDDASDWFAPRYPNEGDPRTAPNEIAFEDPPTTMPSGFELYNDDDDDLSDSGRISYNKKPRTAIGTPVY

Reverse translated and modified with flanking restrictions sites:

GCAGGCGCCAATATTTCAGCCGTGTTCCAGACCTACTATGAGCAGGGGGAGGATGGGCAGGTGTATGAGAGGGGATGTCGGTGGTATGATAAACCCAAGAAGAAGAAGATCTTCCGCTTCGGATCCCACAAGTGGGGAAACGGCGACGGACCAAAGGTGGCTCACAACCTGGGCTTCTACTTCAACGACGACGACGACGACGCCAGCGACTGGTTCGCTCCAAGGTACCCAAACGAGGGCGACCCAAGGACCGCCCCAAACGAGATCGCCTTCGAGGACCCCCCAACCACCATGCCATCCGGCTTCGAGCTGTATAATGACGACGACGACGACCTGAGCGACAGCGGAAGAATCTCCTACAATAAGAAACCAAGAACAGCCATCGGCACCCCAGTCTATGGACGCGTGA

VRP 3

Aa sequence of designed polyepitope (epitopes are underlined):

LSDSGRISYADDGKDPRTAIGTPVYEDGKNEDDQVYERGCRWYEEEDKDEKIFRFGSHKWGNPPQPKVAHNLGFYFDKDDADPPTTMPSGFELYEEGEDPKDASDWFAPRYGNPPQPRTAPNEIAFGEPPDPPNISAVFQTYY

Reverse translated and modified with flanking restrictions sites:

GCAGGCGCCCTCTCCGACTCAGGACGAATCTCTTATGCTGACGACGGGAAGGACCCTAGAACCGCTATTGGAACCCCAGTGTACGAGGATGGAAAAAACGAGGACGACCAGGTGTACGAGAGGGGATGCAGGTGGTACGAGGAGGAGGACAAGGACGAGAAGATCTTCCGCTTCGGCTCCCACAAGTGGGGAAACCCACCACAGCCAAAGGTGGCTCACAACCTGGGCTTCTACTTCGACAAGGACGACGCTGACCCACCAACCACCATGCCATCCGGCTTCGAGCTGTACGAGGAGGGAGAGGACCCAAAGGACGCCAGCGACTGGTTCGCTCCAAGATACGGCAATCCACCACAGCCAAGAACCGCCCCAAACGAGATCGCCTTCGGGGAACCACCAGACCCACCCAATATCTCAGCCGTGTTTCAGACATACTACGGACGCGTGA

VRP 4

Aa sequence of designed polyepitope (epitopes are underlined):

SSEGHLTSVYDDEKPGITANVTDENYEDPQPKVAHNLGFYFDPGDKDRALPFTLSNYDDNEEDYTAQFHPEIFNGPRTAIGTPVYDPGDKDFTWYQLASYQPCDDMVNTTRVTYEGALATAPDGTYNDDDTTMPSGFELYNDDDYAQHMVLSYDDNEEDNSFLDEAAYDPGDKDFVLSWLTPWDPGDKDVRWFAANLLYDGDDLSDSGRISYKPPDGKCVFFLLWRMQQPPPTRARHAIFVYDPGDKDASDWFAPRYKDPKPNISAVFQTYYENVPHSKKDYSFPGPPFFDGDDFLNCAFTFGYPEDPGMPNYHWWVEHDDNEEDQVYERGCRWYNEDDERPFFSSWLVKDPKPMSWRYSCTRYDDEKPGLSASSQTEYDPGDKDIVYSDDLVLY

Reverse translated and modified with flanking restrictions sites:

GCAGGCGCCTCATCCGAAGGCCACCTCACCTCCGTGTACGACGACGAGAAGCCAGGCATCACCGCTAATGTCACCGACGAGAACTACGAAGACCCCCAGCCCAAGGTGGCCCACAACCTGGGCTTCTACTTCGACCCAGGCGACAAGGACCGCGCCCTGCCATTCACCCTGTCCAACTACGACGACAACGAGGAGGACTACACCGCCCAGTTCCACCCCGAGATCTTCAACGGACCAAGGACCGCTATCGGAACCCCCGTGTACGATCCCGGCGACAAGGACTTCACCTGGTACCAGCTGGCCTCCTACCAGCCCTGCGACGACATGGTGAACACCACCCGCGTGACCTACGAGGGAGCCCTGGCTACCGCCCCCGACGGAACCTACAACGACGACGACACCACCATGCCAAGCGGCTTCGAGCTGTACAACGACGACGACTACGCCCAGCACATGGTGCTGTCTTATGATGATAACGAGGAGGACAACAGCTTCCTGGACGAGGCTGCTTATGATCCAGGCGACAAGGACTTCGTGCTGTCCTGGCTGACCCCATGGGATCCTGGCGACAAAGACGTGAGGTGGTTCGCCGCCAACCTGCTGTACGACGGCGACGACCTGTCCGACAGCGGAAGGATCAGCTACAAGCCCCCAGACGGCAAGTGCGTGTTCTTCCTGCTGTGGAGAATGCAGCAGCCACCACCAACCCGCGCTAGGCACGCTATCTTCGTGTACGATCCTGGCGACAAGGACGCCTCCGACTGGTTCGCCCCAAGATACAAGGACCCAAAGCCCAACATCAGCGCCGTGTTCCAGACCTACTACGAGAACGTGCCCCACTCCAAGAAGGACTACAGCTTCCCAGGCCCACCCTTCTTCGACGGCGACGACTTCCTGAACTGCGCCTTCACCTTCGGCTACCCAGAGGACCCCGGCATGCCAAACTACCACTGGTGGGTGGAGCATGATGACAACGAGGAGGACCAGGTGTACGAGAGGGGCTGCAGATGGTACAACGAGGACGACGAGCGGCCCTTCTTCTCCAGCTGGCTGGTGAAGGACCCAAAGCCCATGTCCTGGCGCTACAGCTGCACCCGGTATGATGACGAGAAGCCAGGCCTGAGCGCCTCCAGCCAGACCGAATATGACCCAGGGGATAAAGACATCGTGTACTCAGATGACCTCGTGCTCTACGGACGCGTGA

VRP 5:

Aa sequence of designed polyepitope (epitopes are underlined):

FTWYQLASYEPDDKPYTAQFHPEIFDNDKDLSASSQTEYDDADPERPFFSSWLVEPDDKPNISAVFQTYYNQPDDPTRARHAIFVYDNDKDASDWFAPRYDNDKDLSDSGRISYPPPGNGQVYERGCRWYPDEPGMPNYHWWVEHNQPDDPALATAPDGTYNPKDYAQHMVLSYNPQQPPRTAIGTPVYQEDYSFPGPPFFPPPGNGITANVTDENYDNDKDFLNCAFTFGYPPPQGDIVYSDDLVLYDDADPEVRWFAANLLYNQPDDPRALPFTLSNYDNDKDFVLSWLTPWEPDDKPKVAHNLGFYFDDMVNTTRVTYEPDDKPMSWRYSCTRYDNDKDCVFFLLWRMDNDKDSSEGHLTSVYNPQQPPNSFLDEAAYNQPDDPTTMPSGFELY

Reverse translated and modified with flanking restrictions sites:

GCAGGCGCCTTTACATGGTATCAGCTCGCCTCCTATGAGCCAGACGACAAGCCCTACACCGCCCAGTTTCACCCAGAGATTTTCGACAATGACAAGGACCTGTCCGCCTCCAGCCAGACCGAGTACGACGACGCTGACCCAGAGAGGCCCTTCTTCTCCAGCTGGCTGGTGGAGCCCGACGACAAGCCAAACATCAGCGCCGTGTTCCAGACCTATTATAACCAGCCAGACGACCCAACCCGCGCTAGGCACGCTATCTTCGTGTACGACAACGACAAGGACGCCTCCGACTGGTTCGCCCCCAGATATGATAACGACAAGGACCTGTCCGACAGCGGCAGAATCAGCTACCCACCACCAGGAAACGGACAGGTGTACGAGAGGGGATGCAGATGGTACCCAGACGAGCCCGGCATGCCAAACTACCACTGGTGGGTGGAGCATAATCAGCCCGACGACCCAGCCCTGGCTACCGCCCCCGACGGCACCTACAACCCAAAGGACTACGCCCAGCACATGGTGCTGTCCTACAACCCACAGCAGCCACCAAGGACCGCTATCGGAACCCCCGTGTACCAGGAGGACTACAGCTTCCCAGGACCACCCTTCTTCCCACCACCAGGCAACGGCATCACCGCCAACGTGACCGACGAGAACTATGATAATGACAAGGACTTCCTGAACTGCGCCTTCACCTTCGGCTACCCACCACCACAGGGCGACATCGTGTACTCCGACGACCTGGTGCTGTATGATGACGCTGACCCAGAGGTGCGCTGGTTCGCTGCCAACCTGCTGTACAATCAGCCCGATGACCCACGGGCCCTGCCATTCACCCTGTCCAACTATGATAACGATAAGGACTTCGTGCTGAGCTGGCTGACCCCATGGGAACCCGACGACAAGCCCAAGGTGGCCCACAACCTGGGCTTCTACTTCGACGACATGGTGAACACCACCAGGGTGACCTACGAACCTGACGACAAGCCAATGTCCTGGCGCTACAGCTGCACCCGGTATGATAATGATAAAGACTGCGTGTTCTTCCTGCTGTGGAGAATGGACAACGACAAGGACTCCAGCGAGGGCCACCTGACCTCCGTGTACAACCCCCAGCAGCCACCCAACAGCTTCCTGGACGAAGCCGCCTACAACCAGCCAGACGACCCCACCACAATGCCATCAGGATTTGAACTCTATGGACGCGTGA

VRP 6:

Aa sequence of designed polyepitope (epitopes are underlined):

YAQHMVLSYDDEKPGFTWYQLASYGEDSSEGHLTSVYGGDKNGITANVTDENYDDNEEDYTAQFHPEIFGPDDKPNISAVFQTYYGPDDKPNSFLDEAAYPEDPGMPNYHWWVEHGPDDKPRALPFTLSNYDPGDKDFVLSWLTPWDDADPEVRWFAANLLYGPDDKPQVYERGCRWYEDDDMVNTTRVTYDDGALATAPDGTYGGPRTAIGTPVYDPGDKDIVYSDDLVLYNDPKDFLNCAFTFGYDQEEPASDWFAPRYEDDDYSFPGPPFFDDEKPGCVFFLLWRMPGNEKPRPFFSSWLVQGEPPTTMPSGFELYPEPPTRARHAIFVYQPDPDDLSDSGRISYGPDDKPMSWRYSCTRYQPDPDDLSASSQTEYGPDDKPKVAHNLGFYF

Reverse translated and modified with flanking restrictions sites:

GCAGGCGCCTATGCCCAGCACATGGTCCTGTCCTACGACGATGAGAAGCCCGGATTCACATGGTATCAGCTCGCCAGCTACGGAGAAGATTCCAGCGAGGGACACCTGACCTCCGTGTACGGAGGCGACAAGAACGGCATCACCGCCAACGTGACCGACGAGAACTACGACGACAACGAGGAGGACTACACCGCCCAGTTCCACCCCGAGATCTTCGGCCCAGACGACAAGCCCAACATCTCCGCCGTGTTCCAGACCTACTATGGACCCGATGACAAGCCCAACAGCTTCCTGGACGAGGCTGCTTACCCAGAGGACCCAGGCATGCCCAACTACCACTGGTGGGTGGAGCACGGACCAGACGACAAGCCAAGGGCCCTGCCATTCACCCTGTCCAACTACGACCCCGGCGACAAGGACTTCGTGCTGAGCTGGCTGACCCCATGGGACGACGCTGACCCAGAGGTGAGATGGTTCGCTGCTAACCTGCTGTATGGCCCAGACGACAAGCCACAGGTGTACGAGAGGGGCTGCAGATGGTACGAGGACGACGACATGGTGAACACCACCCGCGTGACCTACGACGACGGAGCCCTGGCTACCGCCCCCGACGGCACCTACGGCGGCCCAAGGACCGCTATCGGAACCCCCGTGTACGACCCAGGCGACAAGGACATCGTGTACTCCGACGACCTGGTGCTGTACAACGACCCCAAGGACTTCCTGAACTGCGCCTTCACCTTCGGCTACGACCAGGAGGAGCCAGCTTCCGACTGGTTCGCTCCACGCTACGAGGACGACGACTACAGCTTCCCAGGACCACCATTCTTCGACGACGAGAAGCCAGGCTGCGTGTTCTTCCTGCTGTGGAGGATGCCCGGCAACGAGAAGCCCAGACCATTCTTCTCCAGCTGGCTGGTGCAGGGAGAGCCACCAACCACCATGCCAAGCGGCTTCGAGCTGTACCCAGAGCCACCAACCCGCGCTAGGCACGCTATCTTCGTGTACCAGCCAGACCCAGACGACCTGTCCGACAGCGGAAGGATCTCCTATGGGCCAGACGACAAGCCAATGTCCTGGAGGTACAGCTGCACCCGGTATCAGCCCGACCCAGACGACCTGAGCGCCTCCAGCCAGACAGAGTATGGACCTGACGATAAACCTAAAGTCGCCCACAACCTCGGATTCTATTTCGGACGCGTGA

VRP 7:

Aa sequence of designed polyepitope (epitopes are underlined):

NISAVFQTYYNEEDKDFTWYQLASYNEEDKDMSWRYSCTRYGPEDPDQVYERGCRWYGPEDPDYAQHMVLSYDDEKPGWGVYSAIETWNEEDKDFVLSWLTPWPPQKPPLSFSYTAQFNEEDKDYTAQFHPEIFPPQKPPLTAALNRNRWDEGPPRTAIGTPVYNEEDKDFLNCAFTFGY

Reverse translated and modified with flanking restrictions sites:

GCAGGCGCCAACATTTCCGCCGTGTTTCAGACCTACTACAACGAGGAGGACAAGGACTTTACATGGTATCAGCTGGCTTCATACAACGAGGAGGACAAGGACATGTCCTGGAGGTACAGCTGCACCAGGTACGGACCAGAGGACCCAGACCAGGTGTACGAGAGGGGATGCAGATGGTATGGCCCAGAGGACCCAGACTACGCTCAGCACATGGTGCTGTCCTACGACGACGAGAAGCCAGGATGGGGCGTGTACAGCGCCATCGAGACCTGGAATGAAGAGGACAAGGACTTCGTGCTGTCCTGGCTGACCCCATGGCCACCACAGAAGCCACCACTGTCCTTCAGCTACACCGCCCAGTTCAACGAAGAAGACAAGGACTACACCGCCCAGTTCCACCCCGAGATCTTCCCCCCCCAGAAGCCACCACTGACCGCCGCCCTGAACCGCAACCGGTGGGACGAGGGCCCCCCACGCACCGCCATCGGCACCCCTGTCTACAACGAGGAGGATAAGGATTTTCTGAACTGTGCTTTTACATTTGGATACGGACGCGTGA

VRP 8:

Aa sequence of designed polyepitope (epitopes are underlined):

YAQHMVLSYDPPDEDLSFSYTAQFPPEDPNISAVFQTYYDPPDEDMSWRYSCTRYEPDEGQVYERGCRWYQEDEDDYTAQFHPEIFDGPKDFTWYQLASYEEKKDGWGVYSAIETWPPEEKNLTAALNRNRWDEEEPRTAIGTPVYPPEDPFLNCAFTFGYDPPDEDFVLSWLTPW

Reverse translated and modified with flanking restrictions sites:

GCAGGCGCCTATGCCCAGCACATGGTCCTCTCCTATGATCCCCCCGACGAAGACCTCTCCTTTTCCTACACCGCTCAGTTTCCTCCAGAAGACCCTAACATCTCCGCCGTGTTCCAGACCTACTACGACCCCCCAGACGAGGACATGTCCTGGCGCTACAGCTGCACCAGGTACGAGCCAGACGAGGGCCAGGTGTACGAGAGGGGCTGCAGATGGTACCAGGAGGACGAGGACGACTACACCGCCCAGTTCCACCCCGAGATCTTCGACGGCCCAAAGGACTTCACCTGGTACCAGCTGGCCTCCTACGAGGAGAAGAAGGACGGCTGGGGCGTGTACAGCGCCATCGAGACCTGGCCCCCAGAGGAGAAGAACCTGACCGCCGCCCTGAACAGGAACAGATGGGACGAGGAGGAGCCAAGGACCGCTATCGGAACCCCAGTGTACCCACCAGAGGACCCCTTCCTGAACTGCGCCTTTACATTTGGATACGACCCACCCGATGAGGACTTTGTGCTGTCTTGGCTGACCCCTTGGGGACGCGTGA

VRP 9:

Aa sequence of designed polyepitope (epitopes are underlined):

FTWYQLASYKDEEEDLSFSYTAQFPEDDEKFVLSWLTPWQNGDPDYAQHMVLSYDDDPEPMSWRYSCTRYGPPPGGQVYERGCRWYPPPKDGWGVYSAIETWGPPPGGLTAALNRNRWDDDPEPRTAIGTPVYGGAKDDYTAQFHPEIFDNKKPNISAVFQTYYDEKKDFLNCAFTFGY

Reverse translated and modified with flanking restrictions sites:

GCAGGCGCCTTCACATGGTATCAGCTGGCTTCATACAAGGACGAGGAGGAGGATCTGTCATTCTCATACACCGCTCAGTTTCCCGAGGACGACGAAAAGTTCGTGCTGTCCTGGCTGACCCCATGGCAGAACGGCGACCCAGACTACGCTCAGCACATGGTGCTGAGCTACGACGACGACCCAGAGCCCATGTCCTGGAGGTACAGCTGCACCAGGTACGGACCACCACCAGGAGGACAGGTGTACGAGAGGGGCTGCAGATGGTACCCACCACCAAAGGACGGATGGGGCGTGTACTCCGCCATCGAGACCTGGGGCCCCCCACCAGGAGGACTGACCGCCGCCCTGAACCGCAACCGGTGGGACGACGACCCAGAGCCAAGGACCGCTATCGGAACCCCCGTGTACGGAGGAGCCAAGGACGACTACACCGCCCAGTTCCACCCAGAGATCTTCGACAACAAGAAGCCCAACATCAGCGCCGTGTTCCAGACCTACTATGACGAGAAGAAGGATTTCCTGAACTGTGCTTTTACATTTGGCTATGGACGCGTGA
