# Supplementary material for: Reduced Virus Load in Lungs of Pigs Challenged with Porcine Reproductive and Respiratory Syndrome Virus after Vaccination with Virus Replicon Particles Encoding Conserved PRRSV Cytotoxic T-Cell Epitopes
Source: Vaccines (Basel). 2021 Mar 2;9(3):208. doi: 10.3390/vaccines9030208 (PMC8000205; doi:10.3390/vaccines9030208)
Supplement: Supplementary file 1 [file vaccines-09-00208-s001.zip › SM files/Supplementary Data 2 - Data on peptide preparation.docx]

**Supplementary Data 2 - Data on peptide preparation.**

| **ID** | **Purity measured**  **by supplier (%)** | **Solvent recommended**  **by supplier** | **Final concentration of**  **non-UPW solvent (% v/v)** |
| --- | --- | --- | --- |
| 2 | 97.7 | UPW | NA |
| 4 | 97.9 | UPW | NA |
| 5 | 92.2 | UPW | NA |
| 7 | 90.0 | UPW | NA |
| 9 | 89.7 | 3% NH3 | 23.0 |
| 10 | 97.7 | UPW | NA |
| 11 | 98.4 | UPW | NA |
| 12 | 91.1 | DMSO | 7.6 |
| 13 | 96.1 | 3% NH3 | 12.0 |
| 17 | 89.6 | UPW | NA |
| 18 | 98.1 | UPW | NA |
| 19 | 97.3 | UPW | NA |
| 21 | 96.7 | UPW | NA |
| 22 | 96.7 | UPW | NA |
| 23 | 92.9 | DMSO | 8.6 |
| 24 | 98.2 | UPW | NA |
| 25 | 96.6 | UPW | NA |
| 27 | 97.4 | UPW | NA |
| 28 | 99.1 | NMP | 2.6 |
| 29 | 96.4 | UPW | NA |
| 30 | 91.4 | NMP | 4.6 |
| 33 | 92.1 | UPW | NA |
| 34 | 92.9 | UPW | NA |
| 36 | 98.9 | UPW | NA |
| 38 | 86.7 | 3% NH3 | 11.5 |
| 39 | 99.6 | UPW | NA |
| 43 | 93.9 | UPW | NA |
| 44 | 96.9 | UPW | NA |
| 46 | 95.3 | UPW | NA |
| 48 | 90.2 | 3% NH3 | 37.5 |
| 49 | 89.5 | NMP | 11.8 |
| 50 | 95.7 | 3% NH3 | 4.6 |
| 53 | 92.4 | UPW | NA |

ID refer to the peptides listed in Table 2. All peptides were dissolved to a concentration of 0.5 mM according to the recommendation from the supplier, and were stored at -20 °C in stock vials of 830 uL and working vials of 40 uL. UPW: ultra pure water, NMP: N-methyl-2-pyrrolidone (analytical grade), 3% NH3: 3% ammonia water – prepared from 1.5 ml 25% NH3 in 8.5 ml UPW, DMSO: dimethyl sulfoxide (analytical grade), NA: “Not Applicable”, since no non-UPW was required as solvent.
