# Supplementary figures and images for: Reduced Virus Load in Lungs of Pigs Challenged with Porcine Reproductive and Respiratory Syndrome Virus after Vaccination with Virus Replicon Particles Encoding Conserved PRRSV Cytotoxic T-Cell Epitopes
Source: Vaccines (Basel). 2021 Mar 2;9(3):208. doi: 10.3390/vaccines9030208 (PMC8000205; doi:10.3390/vaccines9030208)

**Supplementary Data 3 – Flow cytometry results**


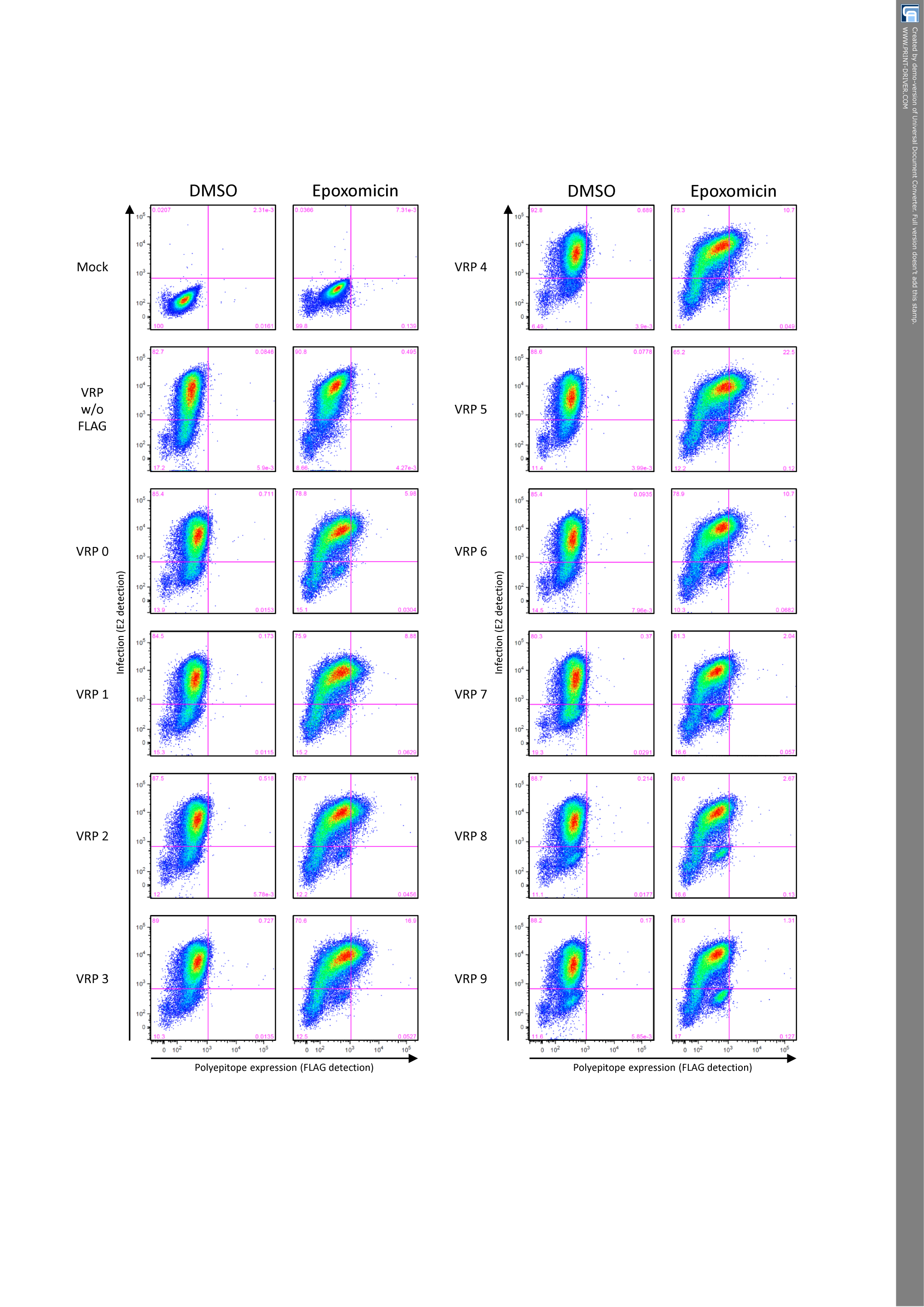

Supplement: Supplementary file 1 [file vaccines-09-00208-s001.zip › SM files/Supplementary Data 3 ¿C Flow cytometry results.docx]
